# Poor Oral Health Linked with Higher Risk of Alzheimer’s Disease

**DOI:** 10.3390/brainsci13111555

**Published:** 2023-11-07

**Authors:** Mihir S. Kulkarni, Brandi C. Miller, Manan Mahani, Rahul Mhaskar, Athanasios Tsalatsanis, Shalini Jain, Hariom Yadav

**Affiliations:** 1 USF Center for Microbiome Research, Microbiomes Institute, Department of Neurosurgery and Brain Repair, University of South Florida, Tampa, FL 33612, USA; 2 USF Center for Microbiome Research, Microbiomes Institute, Department of Molecular Medicine, University of South Florida, Tampa, FL 33612, USA; 3 Department of Internal Medicine, Morsani College of Medicine, University of South Florida, Tampa, FL 33612, USA; 4 Research Methodology and Biostatistics Core, Department of Internal Medicine, Morsani College of Medicine, University of South Florida, Tampa, FL 33612, USA

**Keywords:** Alzheimer’s disease, cognition, dementia, oral health, oral microbiome

## Abstract

Alzheimer’s disease (AD) is a multifactorial neurodegenerative disease characterized by cognitive and behavioral changes in older adults. Emerging evidence suggests poor oral health is associated with AD, but there is a lack of large-scale clinical studies demonstrating this link. Herein, we used the TriNetX database to generate clinical cohorts and assess the risk of AD and survival among >30 million de-identified subjects with normal oral health (*n* = 31,418,814) and poor oral health (*n* = 1,232,751). There was a greater than two-fold increase in AD risk in the poor oral health cohort compared to the normal oral health group (risk ratio (RR): 2.363, (95% confidence interval: 2.326, 2.401)). To reduce potential bias, we performed retrospective propensity score matching for age, gender, and multiple laboratory measures. After matching, the cohorts had no significant differences in survival probability. Furthermore, when comparing multiple oral conditions, diseases related to tooth loss were the most significant risk factor for AD (RR: 3.186, (95% CI: 3.007, 3.376)). Our results suggest that oral health may be important in AD risk, regardless of age, gender, or laboratory measures. However, more large-scale cohort studies are necessary to validate these findings and further evaluate links between oral health and AD.

## 1. Introduction

Alzheimer’s disease (AD) is a progressive neurodegenerative disorder and the most common form of dementia, constituting over 50% of dementia cases in adults 65 years and older [1]. It is characterized by memory loss, decline in cognitive functions, and adverse behavioral changes, which significantly impact quality of life [2]. Globally, it is estimated that over 50 million people are suffering from AD [3], and its prevalence continues to rise. Accumulation of amyloid-β (Aβ) peptides (which aggregate to form neurotoxic plaques) and formation of neurofibrillary tau tangles are two hallmarks of AD pathology, which lead to severe brain and neural atrophy and neurodegeneration [3]. Currently, there are no known preventive or treatment strategies for AD, and this is because we do not fully understand its pathophysiology and risk factors. AD is a multifactorial disease with multiple risk factors, including age, gender, genetics, comorbidities, such as metabolic and cardiovascular diseases, and environmental conditions [2]. However, the magnitude of these risk factors and how they contribute to AD onset or progression remain unknown.

One emerging risk factor of AD is poor oral health, and the current literature suggests a growing link between poor oral health and an increased risk or prevalence of dementia or AD-like pathologies, especially in older adults [4]. Poor oral health often occurs due to inadequate toothbrushing or flossing (poor oral hygiene). It contributes to tooth decay/loss and dental caries or severe oral diseases, such as periodontitis, gingivitis, and even oropharyngeal cancers [5]. However, poor oral health can negatively impact health beyond the oral cavity. Recent cross-sectional and cohort studies have demonstrated that older adults (≥50 years old) with cognitive impairment (CI) or dementia had a significantly higher prevalence of dental caries, poorer oral hygiene, increased mucosal infections (i.e., *Candida*) or lesions, reduced salivary flow, and fewer teeth compared to cognitively healthy controls [6,7,8,9]. Similar findings have been reported in older adults diagnosed with AD [10,11,12,13,14]. Furthermore, tooth loss, dental caries, chronic periodontitis, painful gums, and denture use are also associated with an increased risk of developing dementia or AD [15,16,17]. These studies suggest a link between poor oral health, which contributes to tooth decay or loss or the development of severe oral diseases, and AD progression.

The link between poor oral health and AD has also been reported by assessing changes in the oral microbiota, which is a highly diverse niche of microbes residing in the oral cavity. Furthermore, increased inflammation is a hallmark of many diseases, including oral disease and AD, and is associated with poor oral health. Oral pathogens, such as *Treponema* spirochetes (i.e., *T. pectinovorum* and *T. socranskii*), *Porphyromonas gingivalis*, *Prevotella intermedia*, *Campylobacter rectus*, *Fusobacterium nucleatum*, *Actinomycetales* species, and *Veillonella* species, or their antibodies, are significantly elevated in individuals with AD or are associated with increased prevalence or risk of AD [18,19,20,21]. There is a substantial amount of literature suggesting that infection with *T. pallidum*, which is the causative agent of syphilis and can also present in the oral microbiota, can sustain in the brain, leading to neuroinflammation, chronic infection, and dementia progression [22]. Interestingly, *P. gingivalis* (a keystone pathogen involved in periodontal disease) is linked with accelerated AD pathologies, including CI, Aβ deposition, and inflammatory cytokine production [23].

However, despite these findings, most available studies have been conducted in small cohorts and are localized to specific countries or geographical areas. Thus, there remains a lack of studies demonstrating the link between poor oral health and AD onset or progression in large-scale and diverse cohorts. Elucidating this link on a global scale and in a highly diverse cohort will further highlight the importance of oral health and its contribution to the development of oral diseases and AD, both of which plague the global population. Using a federated research network and database, such as TriNetX, allows for the generation of large, diverse, and effective cohorts from Healthcare Organizations (HCOs) around the globe. These cohorts can be used to investigate and compare disease risks and prevalence.

This study aimed to generate large-scale data from the TriNetX database to determine risk associations and assess survival between poor oral health and AD in individuals 60 years and older. Specifically, we aimed to compare the prevalence of AD between individuals with and without poor oral health and determine which factors contribute to AD risk and survival using propensity score matching. Furthermore, we performed multiple comparisons to determine which oral health conditions are associated with a higher risk of AD.

## 2. Materials and Methods

### 2.1. TriNetX Database

The study was conducted using data from TriNetX, LLC (“TriNetX”), a federated health research network and database. TriNetX provides access to electronic medical records from HCOs worldwide, and the data are publicly available with a login. TriNetX can be accessed at: https://www.trinetx.com (accessed on 4 October 2023). The data used in our study were collected in October 2023 from the TriNetX network, which provides access to electronic medical records, including patient demographics, diagnoses, procedures, medications, laboratory values, and genomic information, from approximately 32 million patients from 79 HCOs. All data were collected per the Health Insurance Portability and Accountability Act (HIPAA). Since this study was retrospective, it was exempt from review by an Institutional Review Board and informed consent was waived. The data generated from the TriNetX platform are a secondary analysis of existing data, do not include any interventions or interactions with human subjects, and are de-identified according to HIPAA privacy rules. TriNetX has the ISO 27001:2013 [24] certification and employs professional teams to protect and maintain patient privacy and data.

### 2.2. Cohorts

**Figure 1:**
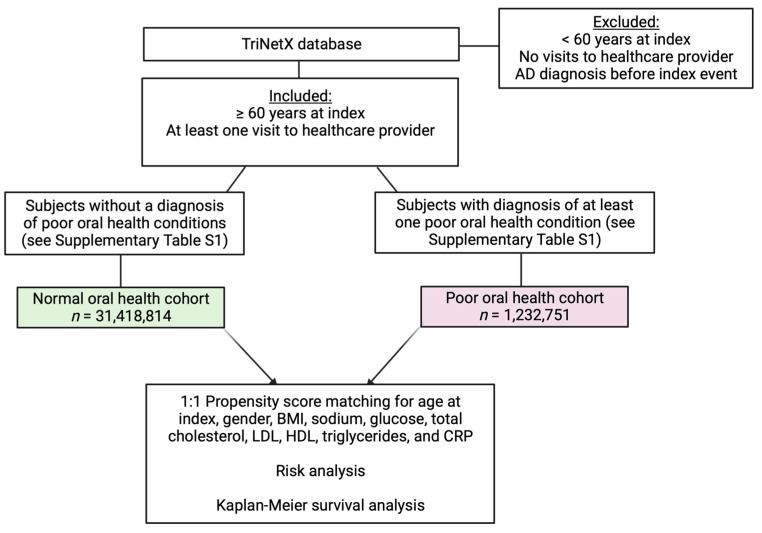
Flow chart for generation of the normal oral health and poor oral health cohorts and analyses performed. AD: Alzheimer’s disease, BMI: body mass index, CRP: C-reactive protein, HDL: high-density lipoprotein cholesterol, LDL: low-density lipoprotein cholesterol.

**Figure 2:**
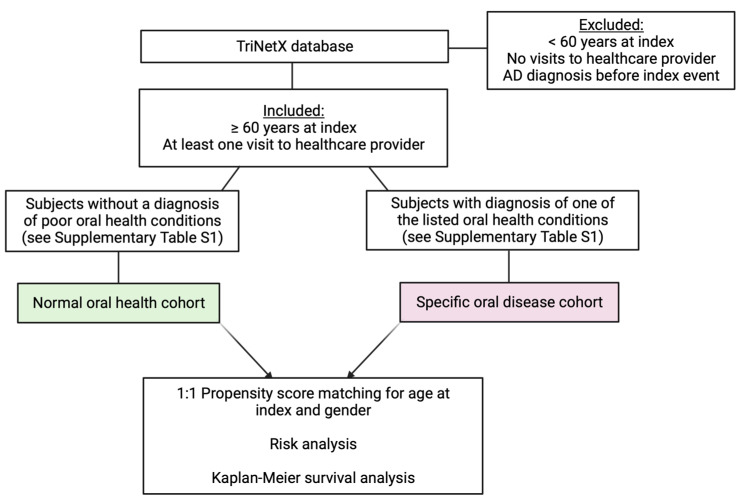
Flow chart for the generation of cohorts for specific oral health conditions and analyses performed. AD: Alzheimer’s disease.

We used the inclusion and exclusion criteria shown in Figure 1 to create the following study cohorts: (1) normal oral health and (2) poor oral health. For both cohorts, we only included patients 60 years or older who had visited a medical office within the last 5 years. The poor oral health cohort was comprised of patients with at least one of the oral health conditions listed in the inclusion criteria (Figure 1), including dental caries, tooth decay or loss, periodontal disease, or gingivitis. These poor oral health conditions have International Code of Diseases (ICDs) identifiers between K00 and K14 in the TriNetX database. Supplementary Table S1 defines these ICDs, which have been established by the World Health Organization. The normal oral health cohort included patients without these oral health conditions. Furthermore, we only included patients who met these criteria no more than five years before the index event. We also generated cohorts for specific oral cavity diseases (ICDs K01–K14) to elucidate which diseases may be associated with increased AD risk and lower survival (Figure 2).

### 2.3. Propensity Score Matching

We first analyzed unmatched cohorts to determine the risk and survival probability between poor oral health and AD. However, because there is likely to be bias due to confounding variables, we performed 1:1 propensity score matching within the TriNetX platform to reduce the effects of these variables. Age and gender are two significant risk factors for AD; as such, we matched the cohorts for age and gender individually and together. Since age and gender are not the only potential confounding variables that could contribute to AD risk, we also matched our data for common laboratory measures that were recorded in TriNetX for exploration purposes only. In particular, we matched for: (1) body mass index (BMI), (2) sodium levels, (3) glucose levels, (4) triglycerides, (5) total cholesterol, (6) low-density lipoprotein (LDL) cholesterol, (7) high-density lipoprotein (HDL) cholesterol, and (8) C-reactive protein (CRP) levels. All measures, except BMI, were reported as concentrations in the whole blood, serum, or plasma. We chose to match these attributes because they were well-recorded among the study cohorts within TriNetX, and they may contribute to obesity and dyslipidemia, which could lead to comorbidities that heighten the risk of AD or lower survival [25]. In particular, these laboratory measures may also be associated with the development of oral diseases or AD.

### 2.4. Statistical Analyses

**Figure 3:**
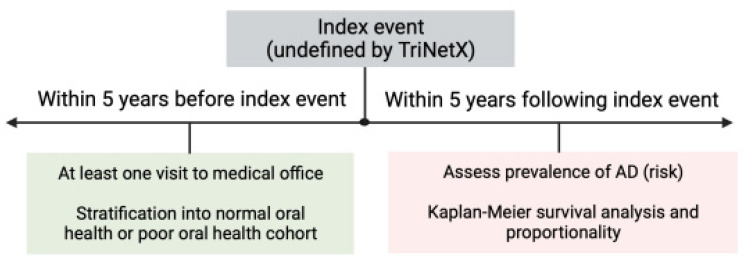
Schematic and timeline depicting when risk and survival analyses were performed in relation to the index event. AD: Alzheimer’s disease.

The incidences of AD (UMLS: ICD10CM:G30) from the normal oral health and poor oral health cohorts within five years of the index event were calculated using risk ratios (RR) and 95% confidence intervals (95% CIs) (Figure 3). The *p*-values were calculated using unpaired *t*-tests to determine differences between the risks. Kaplan–Meier survival analyses were performed to determine survival probabilities across groups to report hazard ratios (HR) with 95% CIs. The log-rank test was used to determine the statistical significance of Kaplan–Meier survival analyses. *p* < 0.05 was considered statistically significant. All statistical analyses were performed using the TriNetX program (TriNetX, Cambridge, MA, USA) in October 2023.

## 3. Results

### 3.1. Generation of Normal Oral Health and Poor Oral Health Cohorts

We generated two cohorts representing 79 HCOs from around the world. After applying inclusion and exclusion criteria, the “normal oral health” cohort (Cohort 1) contained 31,418,814 patients, while the “poor oral health” cohort (Cohort 2) contained 1,232,751 patients. Table 1 shows the demographic data, including age, gender, and race, for both cohorts.

### 3.2. Poor Oral Health Is Associated with an Increased Risk of AD and Decreased Survival Independent of Age, Gender, and Common Laboratory Measures

When analyzing the risk of AD among the two cohorts, we found a greater than two-fold increase in this risk in the poor oral health cohort compared to the normal oral health cohort (RR: 2.36, (95% CI: 2.32, 2.40), *p* < 0.001; Table 2). Kaplan–Meier survival analysis revealed a 98.0% survival probability in the poor oral health group, compared to 98.89% in healthy controls (log-rank *p* < 0.001), with a HR of 1.86 (95% CI: 1.83, 1.89). Together, these findings suggest that poor oral health significantly contributes to AD risk and reduces survival.

However, because AD is a multifactorial disease and can be influenced by many factors, including age, gender, genetics, and comorbidities, it is important to consider that these variables could have contributed to the effect we observed [26]. Therefore, we performed 1:1 propensity score matching to account for the potential bias in our risk and survival analyses. We first matched for age and gender. Table 2 shows the risk and survival analyses for AD between the normal oral health and poor oral health cohorts before matching and after matching for age only, gender only, and age and gender together. Interestingly, the RR, survival probabilities, and HR were similar when the data were unmatched and when matched for age and/or gender (all *p* < 0.001). This suggests that poor oral health significantly increases the risk of AD or reduces survival independent of other risk factors, such as age and gender.

Table 3 shows the risk and survival analyses when matched for age and the eight other attributes one by one. Sodium levels (RR: 2.564, (95% CI: 2.478, 2.653)), blood glucose levels (RR: 2.556, (95% CI: 2.471, 2.644)), and CRP levels (RR: 2.505, (95% CI: 2.431, 2.580)) were associated with the highest risk for AD when matching with age (all *p* < 0.001), but the risk was not significantly higher than in the unmatched cohorts. Furthermore, the HR values for sodium levels (2.241, (95% CI: 2.166, 2.319)), blood glucose levels (2.241, (95% CI: 2.166, 2.318)), and total cholesterol (2.121, (95% CI: 2.053, 2.192)) were the highest in the survival analysis, suggesting they were most associated with an increased risk of mortality within five years. Table 4 shows the risk and survival analyses when matched for gender and our eight chosen laboratory measures. Similarly, blood glucose levels (RR: 2.272, (95% CI: 2.201, 2.346)), blood sodium levels (RR: 2.255, (95% CI: 2.184, 2.328)), and CRP levels (RR: 2.214, (95% CI: 2.152, 2.278)) were the three highest risk factors but were similar to the unmatched data. Additionally, the HR for blood glucose levels (1.966, (95% CI: 1.904, 2.030)), sodium levels (1.942, (95% CI: 1.880, 2.005)), and total cholesterol (1.866, (95% CI: 1.808, 1.926)) were the highest, suggesting reduced survival when matching for these variables with gender. These findings suggest that poor oral health increases the risk of AD more than two-fold, independent of these potential confounding variables, and may also reduce the survival probability. However, other risk factors, such as genetics, socioeconomic status, and education level, may significantly contribute to AD risk, and it is important to consider these and investigate them in future studies.

### 3.3. Different Oral Diseases Are Associated with Varying Risk of AD

Next, we investigated which oral diseases or conditions are associated with an increased risk of AD by further dividing our poor oral health cohort into separate groups. These smaller cohorts were created using the ICD codes for oral diseases published by the World Health Organization (Supplementary Table S1). We matched these cohorts for age and gender to determine if these oral diseases individually increase the risk of AD.

Table 5 compares the risk and survival analyses for specific oral diseases (UMLS:ICD10CM:K01–K14). “Other disorders of teeth and supporting structures” (UMLS:ICD10CM:K08), which includes tooth damage and loss, was associated with a significant and greater than three-fold increase in the risk of AD (RR: 3.186, (95% CI: 3.007, 3.376); *p* < 0.001) and decreased survival (HR: 2.529, (95% CI: 2.386, 2.681); *p* < 0.001). Dental caries (RR: 2.918, (95% CI: 2.728, 3.121); *p* < 0.001), diseases of pulp and periapical tissues (RR: 2.593, (95% CI: 2.389, 2.815); *p* < 0.001), gingivitis and periodontal diseases (RR: 2.823, (95% CI: 2.577, 3.092); *p* < 0.001), and other diseases of lip and oral mucosa (RR: 2.295, (95% CI: 2.166, 2.432); *p* < 0.001) were also associated with a significantly higher risk of AD. Dental caries (HR: 2.358, (95% CI: 2.204, 2.523); *p* < 0.001), diseases of the pulp and periapical tissues (HR: 2.132, (95% CI: 1.963, 2.315); *p* < 0.001), gingivitis and periodontal diseases (HR: 2.195, (95% CI: 2.003, 2.405); *p* = 0.001), and other diseases of lip and oral mucosa (HR: 1.752, (95% CI: 1.653, 1.858); *p* = 0.004) were also linked with reduced survival. These data suggest that specific oral diseases, particularly those that impact the teeth, increase the risk of AD in older adults and may also reduce survival.

## 4. Discussion

This study aimed to determine the risk and survival associations between poor oral health and AD using a large and diverse cohort generated from the TriNetX database. Our findings suggest that poor oral health may significantly increase the risk of AD, and this risk is independent of confounding variables, including age, gender, and common laboratory measures. Furthermore, we found that “other diseases of teeth and supporting structures” (including tooth loss) were associated with the highest risk for AD and significantly reduced survival in our cohorts.

Age and gender are two significant risk factors contributing to the onset and progression of AD. AD prevalence increases with advancing age and has also been linked with gender in previous cohort studies [26,27,28]. Thus, to reduce the potential bias of age and gender, we performed propensity score matching. Interestingly, the risk of AD and survival probabilities were similar when matching for age, gender, or both, compared to our analysis for the unmatched cohorts. Although our data suggest that poor oral health significantly increases AD risk and reduces survival independent of age or gender, it is important to conduct additional studies with large and diverse cohorts to confirm these findings.

Furthermore, we also performed propensity score matching for common laboratory measures, including BMI, sodium, glucose, triglycerides, total cholesterol, LDL cholesterol, HDL cholesterol, and CRP. Although matching for these measures did not significantly change the risk of AD in our poor oral health cohort, it is important to consider abnormalities in these measures as risk factors for AD. For example, low weight (BMI < 20 kg/m^2^) and obesity (BMI > 30 kg/m^2^) have been reported as significant and independent risk factors for AD [29,30,31,32]. Furthermore, hypercholesterolemia has been detected in patients with AD [33] and is linked with Aβ deposition, neurofibrillary tangles, CI, and neuroinflammation in preclinical models [34]. Interestingly, sodium, glucose, and CRP levels were the most significant laboratory measures contributing to AD risk in our poor oral health cohort. Elevated CRP, a pro-inflammatory protein, may increase the risk for AD, especially in individuals with a genetic predisposition [35]. Additionally, chronic elevation of CRP is prevalent in multiple inflammatory comorbidities, including diabetes and cardiovascular diseases [36]. Blood sodium and glucose levels also contribute to conditions such as hypertension (high blood pressure) and type 2 diabetes, which have reported associations with reduced cognitive function and AD pathology (such as weakened blood–brain barrier integrity and Aβ synthesis) [37,38]. In our analyses, we did not consider the prevalence of other comorbidities. However, they are important to address in future cohort studies such as ours because they are (1) linked with poor oral health [39,40,41,42], and (2) linked with an increased risk or prevalence of AD, independent of oral health [43,44].

Kaplan–Meier survival analyses were performed to compare the five-year survival probabilities associated with poor and normal oral health in our cohorts. Although we found significant differences in survival probabilities when comparing poor oral health to normal oral health and some specific oral diseases, our analyses only considered survival for five years. Previous reports align with our study findings, suggesting that poor oral health significantly lowers the survival probability within five years [45,46]. It has also been shown that poor oral health (i.e., periodontal disease) significantly reduces survival probability in older adults (62 years and older) after 10–20 years [47]. Therefore, assessing the survival rate for a longer time window (i.e., ten years or more) could provide even stronger and clinically relevant differences in survival between the two cohorts.

A previous meta-analysis suggested an association between low education and a higher incidence of dementia or AD [48]. Furthermore, lower education or oral health literacy may be linked with less knowledge or implementation of proper oral hygiene practices (i.e., flossing and brushing), more missing teeth, and increased prevalence of dental caries, which could also increase the risk for AD [49,50,51]. Based on these reports, we believe that matching for education level in our data is critical for reducing the potential bias of education on AD risk in our poor oral health cohort. However, although TriNetX does provide patient data regarding education status, including illiteracy or low-level literacy, failed school examinations, underachievement in school, and number of patients having less than a high school diploma (UMLS: ICD10CM Z55.0-Z55.9), these were largely unreported in our cohorts, constituting less than 1% of the poor oral health cohort. Future large-scale cohort studies should be conducted to better elucidate the potential confounding effects of education status on the link between oral health and AD.

In our study, we found that oral diseases classified as “other disorders of teeth and supporting structures” (UMLS: ICD10CM:K08) had the highest risk association with AD, increasing the risk by greater than three-fold compared to healthy controls. According to the ICD codes, many conditions within K08 are related to tooth damage or loss, suggesting that significant tooth damage or loss may be a prominent risk factor for AD [52]. This finding is corroborated by several reports suggesting tooth loss increases the risk for AD or dementia [15,41,53,54,55]. Furthermore, patients with AD reportedly have fewer teeth [56,57]. We also found that dental caries, periodontitis, and gingivitis were significant risk factors for AD in our cohort. Recent studies have also shown that AD is associated with poorer periodontal health and increased prevalence of dental caries [58]. Nonetheless, additional studies are warranted to confirm these findings.

One strength of our study is the large sample size, which makes our data more generalizable. Most previous studies have included smaller sample sizes and analyzed patient data from a specific country or geographical region. Additionally, we used propensity score matching to account for potential confounding variables, such as age, gender, and laboratory measures. This strengthened our data and understanding of the link between poor oral health and AD. Our data indicate that poor oral health is a significant and independent risk factor for AD, but we still lack a full understanding of the mechanisms. It has been suggested that the oral pathogen load and increased inflammation are two major mechanisms contributing to poor oral health, which could heighten the risk for AD [59,60,61]. Therefore, future prospective studies should investigate oral pathogen load and inflammatory markers, such as interleukin (IL)-6, IL-1, and tumor necrosis factor-α, in patients with poor oral health and link them with AD pathologies, such as Aβ accumulation, CI, and neurofibrillary tangle formation. Emerging evidence is also suggesting connections between the oral microbiota and cognitive function [62,63], as well as between the gut microbiota and cognitive function [64,65]. Investigating the oral–gut axis and its effects on cognitive function and AD-like pathologies is also of interest in future studies.

This study has some limitations that should be addressed. First, information from electronic medical records only allows us to make possible correlations based on group analysis, and no specific correlations between individuals can be made. While our data suggest an association between poor oral health and AD, these correlations are limited. Furthermore, this study was retrospective, meaning that the data were previously collected before analysis and that there are likely some absent data. Therefore, it is critical to perform prospective studies in large-scale cohort studies such as ours to validate these findings and reduce potential bias. Lastly, while TriNetX allowed us to perform 1:1 propensity score matching, it only allowed us to match two variables per analysis (i.e., univariate analysis). Therefore, our analyses could have lowered validity because we could not match all confounding variables together. Future studies in large cohorts should use multiple logistic regression analysis to account for all covariates, which will better elucidate the risk association between poor oral health and AD.

## 5. Conclusions

This real-world data analysis showed that poor oral health is associated with an increased risk of AD, with patients with poor oral health demonstrating a 2.363-fold increased risk of AD compared to those in the normal oral health cohort. Interestingly, we found that poor oral health was a risk factor for AD independent of age, gender, and laboratory measures. Furthermore, this study identified that diseases related to tooth loss, dental caries, periodontal diseases, gingivitis, and other diseases of the lip and oral mucosa were associated with a higher risk of AD. Overall, our findings suggest that maintaining or improving healthy oral hygiene and/or oral health may significantly reduce the risk of AD. Future large-scale cohort studies should be conducted to validate our findings and investigate the mechanisms underlying poor oral health and AD pathology.

## Figures and Tables

**Table 1:** Demographics and common laboratories of each cohort before propensity score matching.

Demographic or Laboratory Measure	Cohort 1 (Normal Oral Health)	Cohort 2 (Poor Oral Health)
Number of patients in cohort	31,418,814	1,232,751
Female (%)	16,675,337 (53.1%)	671,281 (54.5%)
Male (%)	14,420,798 (45.9%)	544,851 (44.2%)
Unknown gender or other (%)	322,679 (1.0%)	16,619 (1.3%)
Age (years)	73.9 ± 9.5	72.4 ± 8.9
Race (%)		
White	18,438,027 (58.7%)	792,863 (64.2%)
Black or African American	2,817,690 (9.0%)	176,132 (14.1%)
Asian	815,954 (2.6%)	38,015 (3.7%)
American Indian or Alaska Native	82,909 (0.3%)	3736 (0.3%)
Native Hawaiian/Other Pacific Islander	79,977 (0.3%)	1162 (0.3%)
Unknown race or unreported	9,142,875 (29.1%)	214,499 (17.4%)
BMI (kg/m^2^)	29.1 ± 6.7	28.6 ± 6.5
Blood sodium (mol/vol)	139.0 ± 3.3	139.0 ± 3.5
Blood glucose (mass/vol)	118.0 ± 50.3	123.0 ± 61.2
Triglycerides (mass/vol)	136.0 ± 95.4	142.0 ± 258
Total cholesterol (mass/vol)	177.0 ± 45.1	185.0 ± 46.7
LDL cholesterol (mass/vol)	99.6 ± 37.2	106.0 ± 39.1
HDL cholesterol (mass/vol)	52.5 ± 18.3	52.2 ± 17.6
CRP (mass/vol)	24.0 ± 47.7	30.6 ± 61.0

All laboratory measures (except BMI) were measured in the serum, blood, or plasma and are reported as moles/volume or mass/volume, as indicated. All data are presented as mean ± standard deviation or frequency (%), as appropriate. Abbreviations—BMI: body mass index, CRP: C-reactive protein, HDL: high-density lipoprotein cholesterol, LDL: low-density lipoprotein cholesterol.

**Table 2:** Risk and survival analyses before matching and when matched for age and gender.

Variable(s) Matched	*n*	Patients with AD	Risk	Risk Difference (*p*-Value)	Risk Ratio (95% CI)	Kaplan–Meier Survivability (%)	*p*-Value (Log-Rank Test)	Hazard Ratio (95% CI)	*p*-Value
**None**							*p* < 0.001 *		*p* < 0.001 *
Cohort 1	31,418,814	179,136	0.006	0.008	2.363	98.89%		1.868	
Cohort 2	1,232,751	16,609	0.013	(*p* < 0.001 *)	(2.326, 2.401)	98.00%		(1.838, 1.898)	
**Age only**							*p* < 0.001 *		*p* < 0.001 *
Cohort 1	1,233,771	6864	0.006	0.008	2.489	98.92%		1.975	
Cohort 2	1,233,771	17,083	0.014	(*p* < 0.001 *)	(2.420, 2.559)	97.95%		(1.921, 2.032)	
**Gender only**							*p* < 0.001 *		*p* < 0.001 *
Cohort 1	1,221,975	7235	0.006	0.008	2.318	98.85%		1.834	
Cohort 2	1,221,975	16,774	0.014	(*p* < 0.001 *)	(2.256, 2.383)	97.96%		(1.784, 1.885)	
**Age and gender**							*p* < 0.001 *		*p* < 0.001 *
Cohort 1	1,231,244	6784	0.006	0.008	2.508	98.94%		1.995	
Cohort 2	1,231,244	17,017	0.014	(*p* < 0.001 *)	(2.439, 2.580)	97.96%		(1.939, 2.052)	

Cohort 1 represents the “normal oral health” cohort, while Cohort 2 represents the “poor oral health” cohort. Propensity score matching was performed to match for age and gender. Statistically significant risks or survival probabilities are indicated by an asterisk. Abbreviations—AD: Alzheimer’s disease, 95% CI: 95% confidence interval.

**Table 3:** Risk and survival analyses when matched for age and laboratory measures.

Measure Matched with Age	*n*	Patients with AD	Risk	Risk Difference (*p*-Value)	Risk Ratio (95% CI)	Kaplan–Meier Survivability (%)	*p*-Value (Log-Rank Test)	Hazard Ratio (95% CI)	*p*-Value
**BMI (kg/m^2^)**							*p* < 0.001 *		*p* < 0.001 *
Cohort 1	919,405	5161	0.006	0.007	2.289	98.93%		1.860	
Cohort 2	919,405	11,813	0.013	(*p* < 0.001 *)	(2.216, 2.365)	98.06%		(1.800, 1922)	
**Sodium (mmol/L)**							*p* < 0.001 *		*p* < 0.001 *
Cohort 1	951,651	4551	0.005	0.007	2.564	99.15%		2.241	
Cohort 2	951,651	11,670	0.012	(*p* < 0.001 *)	(2.478, 2.653)	98.16%		(2.166, 2.319)	
**Glucose (mg/dL)**							*p* < 0.001 *		*p* < 0.001 *
Cohort 1	951,822	4632	0.005	0.008	2.556	99.14%		2.241	
Cohort 2	951,822	11,838	0.012	(*p* < 0.001 *)	(2.471, 2.644)	98.13%		(2.166, 2.318)	
**Triglycerides (mg/dL)**							*p* < 0.001 *		*p* < 0.001 *
Cohort 1	979,884	5085	0.005	0.007	2.422	99.07%		2.118	
Cohort 2	979,884	12,315	0.013	(*p* < 0.001 *)	(2.344, 2.502)	98.10%		(2.050, 2.189)	
**Cholesterol (mg/dL)**							*p* < 0.001 *		*p* < 0.001 *
Cohort 1	974,220	5078	0.005	0.007	2.413	99.06%		2.121	
Cohort 2	974,220	12,254	0.013	(*p* < 0.001 *)	(2.336, 2.493)	98.09%		(2.053, 2.192)	
**LDL (mg/dL)**							*p* < 0.001 *		*p* < 0.001 *
Cohort 1	973,827	5089	0.005	0.007	2.390	99.06%		2.100	
Cohort 2	973,827	12,165	0.012	(*p* < 0.001 *)	(2.314, 2.470)	98.10%		(2.032, 2.170)	
**HDL (mg/dL)**							*p* < 0.001 *		*p* < 0.001 *
Cohort 1	952,564	4941	0.005	0.007	2.387	99.07%		2.105	
Cohort 2	952,564	11,794	0.012	(*p* < 0.001 *)	(2.309, 2.467)	98.12%		(2.036, 2.176)	
**CRP (mg/dL)**							*p* < 0.001 *		*p* < 0.001 *
Cohort 1	1,116,404	6001	0.005	0.008	2.505	98.96%		2.000	
Cohort 2	1,116,404	15,030	0.013	(*p* < 0.001 *)	(2.431, 2.580)	98.01%		(1.941, 2.060)	

Cohort 1 represents the “normal oral health” cohort, while Cohort 2 represents the “poor oral health” cohort. Propensity score matching was performed to match for age and common laboratory measures. Statistically significant risks or survival probabilities are indicated by an asterisk. Abbreviations—AD: Alzheimer’s disease, BMI: body mass index, CRP: C-reactive protein, HDL: high-density lipoprotein cholesterol, LDL: low-density lipoprotein cholesterol, 95% CI: 95% confidence interval.

**Table 4:** Risk and survival analyses after matching for gender and laboratory measures.

Measure Matched with Gender	*n*	Patients with AD	Risk	Risk Difference (*p*-Value)	Risk Ratio (95% CI)	Kaplan–Meier Survivability (%)	*p*-Value (Log-Rank Test)	Hazard Ratio (95% CI)	*p*-Value
**BMI (kg/m^2^)**							*p* < 0.001 *		*p* < 0.001 *
Cohort 1	921,644	5919	0.006	0.006	2.011	98.76%		1.617	
Cohort 2	921,644	11,904	0.013	(*p* < 0.001 *)	(1.950, 2.075)	98.05%		(1.567, 1.668)	
**Sodium (mmol/L)**							*p* < 0.001 *		*p* < 0.001 *
Cohort 1	952,388	5384	0.006	0.007	2.255	98.98%		1.942	
Cohort 2	952,388	12,142	0.013	(*p* < 0.001 *)	(2.184, 2.328)	98.08%		(1.880, 2.005)	
**Glucose (mg/dL)**							*p* < 0.001 *		*p* < 0.001 *
Cohort 1	952,489	5399	0.006	0.007	2.272	98.98%		1.966	
Cohort 2	952,489	12,267	0.013	(*p* < 0.001 *)	(2.201, 2.346)	98.06%		(1.904, 2.030)	
**Triglycerides (mg/dL)**							*p* < 0.001 *		*p* < 0.001 *
Cohort 1	958,532	5649	0.006	0.007	2.145	98.94%		1.860	
Cohort 2	958,532	12,116	0.013	(*p* < 0.001 *)	(2.078, 2.213)	98.09%		(1.802, 1.920)	
**Cholesterol (mg/dL)**							*p* < 0.001 *		*p* < 0.001 *
Cohort 1	955,709	5636	0.005	0.007	2.138	98.94%		1.866	
Cohort 2	955,709	12,050	0.013	(*p* < 0.001 *)	(2.072, 2.206)	98.08%		(1.808, 1.926)	
**LDL (mg/dL)**							*p* < 0.001 *		*p* < 0.001 *
Cohort 1	953,687	5645	0.006	0.007	2.116	98.93%		1.845	
Cohort 2	953,687	11,945	0.013	(*p* < 0.001 *)	(2.050, 2.184)	98.10%		(1.787, 1.904)	
**HDL (mg/dL)**							*p* < 0.001 *		*p* < 0.001 *
Cohort 1	953,537	5622	0.006	0.007	2.136	98.94%		1.861	
Cohort 2	953,537	12,006	0.013	(*p* < 0.001 *)	(2.069, 2.204)	98.09%		(1.803, 1.921)	
**CRP (mg/dL)**							*p* < 0.001 *		*p* < 0.001 *
Cohort 1	1,118,368	6819	0.006	0.007	2.214	98.82%		1.753	
Cohort 2	1,118,368	15,099	0.014	(*p* < 0.001 *)	(2.152, 2.278)	98.00%		(1.704, 1.804)	

Cohort 1 represents the “normal oral health” cohort, while Cohort 2 represents the “poor oral health” cohort. Propensity score matching was performed to match for gender and common laboratory measures. Statistically significant risks or survival probabilities are indicated by an asterisk. Abbreviations—AD: Alzheimer’s disease, BMI: body mass index, CRP: C-reactive protein, HDL: high-density lipoprotein cholesterol, LDL: low-density lipoprotein cholesterol, 95% CI: 95% confidence interval.

**Table 5:** Risk and survival analyses for specific diseases of the oral cavity.

Oral Disease or Condition	*n*	Patients with AD	Risk	Risk Difference (*p*-Value)	Risk Ratio (95% CI)	Kaplan–Meier Survivability (%)	*p*-Value (Log-Rank Test)	Hazard Ratio (95% CI)	*p*-Value
UMLS:ICD10CM:K01							*p* = 0.215		*p* = 0.215
**Embedded and impacted teeth**									
Cohort 1	13,922	58	0.004	0.002	1.517	99.25%		1.233	
Cohort 2	13,922	88	0.006	(*p* = 0.013 *)	(1.090, 2.112)	99.12%		(0.885, 1.718)	
UMLS:ICD10CM:K02							*p* < 0.001 *		*p* < 0.001 *
**Dental caries**									
Cohort 1	270,176	1134	0.004	0.008	2.918	99.23%		2.358	
Cohort 2	270,176	3309	0.012	(*p* < 0.001 *)	(2.728, 3.121)	98.25%		(2.204, 2.523)	
UMLS:ICD10CM:K03							*p* < 0.001 *		*p* = 0.313
**Other diseases of hard tissues of teeth**									
Cohort 1	43,765	190	0.004	0.008	2.684	99.20%		2.056	
Cohort 2	43,765	510	0.012	(*p* < 0.001 *)	(2.274, 3.169)	98.39%		(1.740, 2.428)	
UMLS:ICD10CM:K04							*p* < 0.001 *		*p* < 0.001 *
**Diseases of pulp and periapical tissues**									
Cohort 1	177,872	787	0.004	0.007	2.593	99.17%		2.132	
Cohort 2	177,872	2041	0.011	(*p* < 0.001 *)	(2.389, 2.815)	98.31%		(1.963, 2.315)	
UMLS:ICD10CM:K05							*p* < 0.001 *		*p* = 0.001 *
**Gingivitis and periodontal diseases**									
Cohort 1	138,077	621	0.004	0.009	2.823	99.14%		2.195	
Cohort 2	138,077	1753	0.013	(*p* < 0.001 *)	(2.577, 3.092)	98.21%		(2.003, 2.405)	
UMLS:ICD10CM:K06							*p* < 0.001 *		*p* = 0.376
**Other diseases of gingiva and edentulous alveolar ridge**									
Cohort 1	32,500	204	0.006	0.010	2.637	98.76%		2.066	
Cohort 2	32,500	538	0.017	(*p* < 0.001 *)	(2.246, 3.096)	97.53%		(1.758, 2.428)	
UMLS:ICD10CM:K08							*p* < 0.001 *		*p* < 0.001 *
**Other disorders of teeth and supporting structures**									
Cohort 1	300,038	1493	0.005	0.011	3.186	99.07%		2.529	
Cohort 2	300,038	4757	0.016	(*p* < 0.001 *)	(3.007, 3.376)	97.72%		(2.386, 2.681)	
UMLS:ICD10CM:K09							*p* = 0.061		*p* = 0.465
**Cysts of oral region, not elsewhere classified**									
Cohort 1	9954	50	0.005	0.004	1.760	99.05%		1.392	
Cohort 2	9954	88	0.009	(*p* = 0.001 *)	(1.245, 2.488)	98.65%		(0.984, 1.970)	
UMLS:ICD10CM:K11							*p* < 0.001 *		*p* = 0.347
**Diseases of salivary glands**									
Cohort 1	236,672	1596	0.007	0.008	2.283	98.66%		1.772	
Cohort 2	236,672	3644	0.015	(*p* < 0.001 *)	(2.153, 2.421)	97.67%		(1.671, 1.879)	
UMLS:ICD10CM:K12							*p* < 0.001 *		*p* = 0.096
**Stomatitis and related lesions**									
Cohort 1	215,892	1168	0.005	0.006	2.115	98.95%		1.684	
Cohort 2	215,892	2470	0.011	(*p* < 0.001 *)	(1.973, 2.267)	98.26%		(1.571, 1.806)	
UMLS:ICD10CM:K13							*p* < 0.001 *		*p* = 0.004 *
**Other diseases of lip and oral mucosa**									
Cohort 1	267,285	1628	0.006	0.008	2.295	98.93%		1.752	
Cohort 2	267,285	3737	0.014	(*p* < 0.001 *)	(2.166, 2.432)	97.93%		(1.653, 1.858)	
UMLS:ICD10CM:K14							*p* < 0.001 *		*p* = 0.726
**Diseases of tongue**									
Cohort 1	119,952	733	0.006	0.007	2.097	98.78%		1.617	
Cohort 2	119,952	1537	0.013	(*p* < 0.001 *)	(1.921, 2.289)	98.02%		(1.481, 1.766)	

Cohort 1 represents the “normal oral health” cohort, while Cohort 2 represents the “poor oral health” cohort. Propensity score matching was performed to match for age and gender for all comparisons. Statistically significant risks or survival probabilities are indicated by an asterisk. Abbreviations—AD: Alzheimer’s disease, 95% CI: 95% confidence interval.

## Data Availability

Data generated in this study are publicly available from the TriNetX database at: http://www.trinetx.com (accessed on 4 October 2023).

## Supplementary Materials

The following supporting information can be downloaded at: https://www.mdpi.com/article/10.3390/brainsci13111555/s1, Table S1: International Codes of Diseases for Diseases of the Oral Cavity and Salivary Glands.

## References

[1] Kumar A., Sidhu J., Goyal A., Tsao J.W., Doerr C. (2021). Alzheimer Disease (Nursing).

[2] Breijyeh Z., Karaman R. (2020). Comprehensive review on Alzheimer’s disease: Causes and treatment. Molecules.

[3] DeTure M.A., Dickson D.W. (2019). The neuropathological diagnosis of Alzheimer’s disease. Mol. Neurodegener..

[4] Hamza S.A., Asif S., Bokhari S.A.H. (2021). Oral health of individuals with dementia and Alzheimer’s disease: A review. J. Indian Soc. Periodontol..

[5] Petersen P.E., Bourgeois D., Ogawa H., Estupinan-Day S., Ndiaye C. (2005). The global burden of oral diseases and risks to oral health. Bull. World Health Organ..

[6] Gao S.S., Chen K.J., Duangthip D., Lo E.C.M., Chu C.H. (2020). The oral health status of Chinese elderly people with and without dementia: A cross-sectional study. Int. J. Environ. Res. Public Health.

[7] Linda S.K., Tri B.R., Dinni A., Chaidar M., Sri L., Eef H. (2015). Oral hygiene status and cognitive function in Indonesian elderly. Int. J. Clin. Prev. Dent..

[8] Saito S., Ohi T., Murakami T., Komiyama T., Miyoshi Y., Endo K., Satoh M., Asayama K., Inoue R., Kikuya M. (2018). Association between tooth loss and cognitive impairment in community-dwelling older Japanese adults: A 4-year prospective cohort study from the Ohasama study. BMC Oral Health.

[9] Ranjan R., Rout M., Mishra M., Kore S.A. (2019). Tooth loss and dementia: An oro-neural connection. A cross-sectional study. J. Indian Soc. Periodontol..

[10] Martande S.S., Pradeep A., Singh S.P., Kumari M., Suke D.K., Raju A.P., Naik S.B., Singh P., Guruprasad C., Chatterji A. (2014). Periodontal health condition in patients with Alzheimer’s disease. Am. J. Alzheimers Dis. Other Demen..

[11] D’Alessandro G., Costi T., Alkhamis N., Bagattoni S., Sadotti A., Piana G. (2018). Oral Health Status in Alzheimer’s Disease Patients: A Descriptive Study in an Italian Population. J. Contemp. Dent. Pract..

[12] Holmer J., Eriksdotter M., Schultzberg M., Pussinen P.J., Buhlin K. (2018). Association between periodontitis and risk of Alzheimer’s disease, mild cognitive impairment and subjective cognitive decline: A case–control study. J. Clin. Periodontol..

[13] Aragón F., Zea-Sevilla M., Montero J., Sancho P., Corral R., Tejedor C., Frades-Payo B., Paredes-Gallardo V., Albaladejo A. (2018). Oral health in Alzheimer’s disease: A multicenter case-control study. Clin. Oral Investig..

[14] Ide M., Harris M., Stevens A., Sussams R., Hopkins V., Culliford D., Fuller J., Ibbett P., Raybould R., Thomas R. (2016). Periodontitis and cognitive decline in Alzheimer’s disease. PLoS ONE.

[15] Lee K.H., Choi Y.Y. (2019). Association between oral health and dementia in the elderly: A population-based study in Korea. Sci. Rep..

[16] Chen C.-K., Wu Y.-T., Chang Y.-C. (2017). Association between chronic periodontitis and the risk of Alzheimer’s disease: A retrospective, population-based, matched-cohort study. Alzheimers Res. Ther..

[17] Zhang S., Yang F., Wang Z., Qian X., Ji Y., Gong L., Ge S., Yan F. (2020). Poor oral health conditions and cognitive decline: Studies in humans and rats. PLoS ONE.

[18] Riviere G.R., Riviere K.H., Smith K.S. (2002). Molecular and immunological evidence of oral Treponema in the human brain and their association with Alzheimer’s disease. Oral Microbiol. Immunol..

[19] Kamer A.R., Craig R.G., Pirraglia E., Dasanayake A.P., Norman R.G., Boylan R.J., Nehorayoff A., Glodzik L., Brys M., de Leon M.J. (2009). TNF-α and antibodies to periodontal bacteria discriminate between Alzheimer’s disease patients and normal subjects. J. Neuroimmunol..

[20] Beydoun M.A., Beydoun H.A., Weiss J., Hossain S., El-Hajj Z.W., Zonderman A.B. (2021). Helicobacter pylori, periodontal pathogens, and their interactive association with incident all-cause and Alzheimer’s disease dementia in a large national survey. Mol. Psychiatry.

[21] Siddiqui H., Eribe E.R.K., Singhrao S.K., Olsen I. (2019). High throughput sequencing detects gingivitis and periodontal oral bacteria in Alzheimer’s disease autopsy brains. J. Neurosci. Res..

[22] Miklossy J. (2015). Historic evidence to support a causal relationship between spirochetal infections and Alzheimer’s disease. Front. Aging Neurosci..

[23] Ishida N., Ishihara Y., Ishida K., Tada H., Funaki-Kato Y., Hagiwara M., Ferdous T., Abdullah M., Mitani A., Michikawa M. (2017). Periodontitis induced by bacterial infection exacerbates features of Alzheimer’s disease in transgenic mice. NPJ Aging Mech. Dis..

[24] (2013). Information Technology—Security Techniques—Information Security Management Systems—Requirements.

[25] Silva M.V.F., Loures C.D.M.G., Alves L.C.V., de Souza L.C., Borges K.B.G., Carvalho M.d.G. (2019). Alzheimer’s disease: Risk factors and potentially protective measures. J. Biomed. Sci..

[26] Armstrong R.A. (2019). Risk factors for Alzheimer’s disease. Folia Neuropathol..

[27] Andersen K., Launer L.J., Dewey M.E., Letenneur L., Ott A., Copeland J., Dartigues J.-F., Kragh–Sorensen P., Baldereschi M., Brayne C. (1999). Gender differences in the incidence of AD and vascular dementia: The EURODEM Studies. Neurology.

[28] Lobo A., Launer L.J., Fratiglioni L., Andersen K., Di Carlo A., Breteler M., Copeland J., Dartigues J., Jagger C., Martinez-Lage J. (2000). Prevalence of dementia and major subtypes in Europe: A collaborative study of population-based cohorts. Neurology.

[29] Profenno L.A., Porsteinsson A.P., Faraone S.V. (2010). Meta-analysis of Alzheimer’s disease risk with obesity, diabetes, and related disorders. Biol. Psychiatry.

[30] Fitzpatrick A.L., Kuller L.H., Lopez O.L., Diehr P., O’Meara E.S., Longstreth W., Luchsinger J.A. (2009). Midlife and late-life obesity and the risk of dementia: Cardiovascular health study. Arch. Neurol..

[31] Anstey K., Cherbuin N., Budge M., Young J. (2011). Body mass index in midlife and late-life as a risk factor for dementia: A meta-analysis of prospective studies. Obes. Rev..

[32] Qizilbash N., Gregson J., Johnson M.E., Pearce N., Douglas I., Wing K., Evans S.J., Pocock S.J. (2015). BMI and risk of dementia in two million people over two decades: A retrospective cohort study. Lancet Diabetes Endocrinol..

[33] Popp J., Meichsner S., Kölsch H., Lewczuk P., Maier W., Kornhuber J., Jessen F., Lütjohann D. (2013). Cerebral and extracerebral cholesterol metabolism and CSF markers of Alzheimer’s disease. Biochem. Pharmacol..

[34] Ullrich C., Pirchl M., Humpel C. (2010). Hypercholesterolemia in rats impairs the cholinergic system and leads to memory deficits. Mol. Cell. Neurosci..

[35] Huang J., Tao Q., Ang T.F.A., Farrell J., Zhu C., Wang Y., Stein T.D., Lunetta K.L., Massaro J., Mez J. (2022). The impact of increasing levels of blood C-reactive protein on the inflammatory loci SPI1 and CD33 in Alzheimer’s disease. Transl. Psychiatry.

[36] Sproston N.R., Ashworth J.J. (2018). Role of C-reactive protein at sites of inflammation and infection. Front. Immunol..

[37] Skoog I., Lernfelt B., Landahl S., Palmertz B., Andreasson L.A., Nilsson L., Persson G., Oden A., Svanborg A. (1996). 15-year longitudinal study of blood pressure and dementia. Lancet.

[38] Kimura N. (2016). Diabetes mellitus induces Alzheimer’s disease pathology: Histopathological evidence from animal models. Int. J. Mol. Sci..

[39] Laouali N., Fatouhi D.E., Aguayo G., Balkau B., Boutron-Ruault M.-C., Bonnet F., Fagherazzi G. (2021). Type 2 diabetes and its characteristics are associated with poor oral health: Findings from 60,590 senior women from the E3N study. BMC Oral Health.

[40] Han S.-J., Son Y.-J., Kim B.-H. (2021). Association between diabetes mellitus and oral health status in patients with cardiovascular diseases: A nationwide population-based study. Int. J. Environ. Res. Public Health.

[41] Li L., Zhang Q., Yang D., Yang S., Zhao Y., Jiang M., Wang X., Zhao L., Liu Q., Lu Z. (2023). Tooth loss and the risk of cognitive decline and dementia: A meta-analysis of cohort studies. Front. Neurol..

[42] Pietropaoli D., Pinto R.D., Ferri C., Wright J.T., Giannoni M., Ortu E., Monaco A. (2018). Poor oral health and blood pressure control among US hypertensive adults: Results from the national health and nutrition examination survey 2009 to 2014. Hypertension.

[43] Athanasaki A., Melanis K., Tsantzali I., Stefanou M.I., Ntymenou S., Paraskevas S.G., Kalamatianos T., Boutati E., Lambadiari V., Voumvourakis K.I. (2022). Type 2 diabetes mellitus as a risk factor for Alzheimer’s disease: Review and meta-analysis. Biomedicines.

[44] Leszek J., Mikhaylenko E.V., Belousov D.M., Koutsouraki E., Szczechowiak K., Kobusiak-Prokopowicz M., Mysiak A., Diniz B.S., Somasundaram S.G., Kirkland C.E. (2021). The links between cardiovascular diseases and Alzheimer’s disease. Curr. Neuropharmacol..

[45] Farquhar D.R., Divaris K., Mazul A.L., Weissler M.C., Zevallos J.P., Olshan A.F. (2017). Poor oral health affects survival in head and neck cancer. Oral Oncol..

[46] Kotronia E., Brown H., Papacosta A.O., Lennon L.T., Weyant R.J., Whincup P.H., Wannamethee S.G., Ramsay S.E. (2021). Oral health and all-cause, cardiovascular disease, and respiratory mortality in older people in the UK and USA. Sci. Rep..

[47] Demmer R.T., Norby F.L., Lakshminarayan K., Walker K.A., Pankow J.S., Folsom A.R., Mosley T., Beck J., Lutsey P.L. (2020). Periodontal disease and incident dementia: The Atherosclerosis Risk in Communities Study (ARIC). Neurology.

[48] Xu W., Tan L., Wang H.-F., Tan M.-S., Tan L., Li J.-Q., Zhao Q.-F., Yu J.-T. (2016). Education and risk of dementia: Dose-response meta-analysis of prospective cohort studies. Mol. Neurobiol..

[49] Batista M.J., Lawrence H.P., Sousa M.D.L.R.D. (2018). Oral health literacy and oral health outcomes in an adult population in Brazil. BMC Public Health.

[50] Cepova E., Cicvakova M., Kolarcik P., Markovska N., Geckova A.M. (2018). Associations of multidimensional health literacy with reported oral health promoting behaviour among Slovak adults: A cross-sectional study. BMC Oral Health.

[51] Márquez-Arrico C.-F., Almerich-Silla J.-M., Montiel-Company J.-M. (2019). Oral health knowledge in relation to educational level in an adult population in Spain. J. Clin. Exp. Dent..

[52] Weatherspoon D., Chattopadhyay A. (2013). International classification of diseases codes and their use in dentistry. J. Dent. Oral Craniofac. Epidemiol..

[53] Fang W.-L., Jiang M.-J., Gu B.-B., Wei Y.-M., Fan S.-N., Liao W., Zheng Y.-Q., Liao S.-W., Xiong Y., Xiao S.-H. (2018). Tooth loss as a risk factor for dementia: Systematic review and meta-analysis of 21 observational studies. BMC Psychiatry.

[54] Asher S., Stephen R., Mantyla P., Suominen A.L., Solomon A. (2022). Periodontal health, cognitive decline, and dementia: A systematic review and meta-analysis of longitudinal studies. J. Am. Geriatr. Soc..

[55] Tsuneishi M., Yamamoto T., Yamaguchi T., Kodama T., Sato T. (2021). Association between number of teeth and Alzheimer’s disease using the National Database of Health Insurance Claims and Specific Health Checkups of Japan. PLoS ONE.

[56] Dioguardi M., Gioia G.D., Caloro G.A., Capocasal G., Zhurakivska K., Troiano G., Russo L.L., Muzio L.L. (2019). The association between tooth loss and Alzheimer’s disease: A systematic review with meta-analysis of case control studies. Dent. J..

[57] Yoo J.-J., Yoon J.-H., Kang M.-J., Oh N. (2019). The effect of missing teeth on dementia in older people: A nationwide population-based cohort study in South Korea. BMC Oral Health.

[58] Nadim R., Tang J., Dilmohamed A., Yuan S., Wu C., Bakre A.T., Partridge M., Ni J., Copeland J.R., Antsey K.J. (2020). Influence of periodontal disease on risk of dementia: A systematic literature review and a meta-analysis. Eur. J. Epidemiol..

[59] Delaby C., Hirtz C., Lehmann S. (2023). Overview of the blood biomarkers in Alzheimer’s disease: Promises and challenges. Rev. Neurol..

[60] Sadrameli M., Bathini P., Alberi L. (2020). Linking mechanisms of periodontitis to Alzheimer’s disease. Curr. Opin. Neurol..

[61] Cestari J.A.F., Fabri G.M.C., Kalil J., Nitrini R., Jacob-Filho W., de Siqueira J.T.T., Siquiera S.R.D.T. (2016). Oral infections and cytokine levels in patients with Alzheimer’s disease and mild cognitive impairment compared with controls. J. Alzheimers Dis..

[62] Liu S., Dashper S.G., Zhao R. (2023). Association between oral bacteria and Alzheimer’s disease: A systematic review and meta-analysis. J. Alzheimers Dis..

[63] Guo H., Li B., Yao H., Liu D., Chen R., Zhou S., Ji Y., Zeng L., Du M. (2023). Profiling the oral microbiomes in patients with Alzheimer’s disease. Oral Dis..

[64] Chaudhari D.S., Jain S., Yata V.K., Mishra S.P., Kumar A., Fraser A., Kociolek J., Dangiolo M., Smith A., Golden A. (2023). Unique trans-kingdom microbiome structural and functional signatures predict cognitive decline in older adults. Geroscience.

[65] Nagpal R., Neth B.J., Wang S., Craft S., Yadav H. (2019). Modified Mediterranean-ketogenic diet modulates gut microbiome and short-chain fatty acids in association with Alzheimer’s disease markers in subjects with mild cognitive impairment. EBioMedicine.

