# Supplementary material for: Poor Oral Health Linked with Higher Risk of Alzheimer’s Disease
Source: Brain Sci. 2023 Nov 7;13(11):1555. doi: 10.3390/brainsci13111555 (PMC10669972; doi:10.3390/brainsci13111555)
Supplement: Supplementary file 1 [file brainsci-13-01555-s001.zip › brainsci-2698014-supplementary.pdf]

brain sciences

Table S1. International Codes of Diseases for Diseases of the Oral Cavity and Salivary Glands.

ICD-CM Code	Disease Name
**UMLS:ICD 10CM:K00**	**Disorders of tooth development**
K00.0	Anodontia
K00.1	Supernumerary teeth
K00.2	Abnormalities of size and form of teeth
K00.3	Mottled teeth
K00.4	Disturbances in tooth formation
K00.5	Hereditary disturbances in tooth structure, not elsewhere classified
K00.6	Disturbances in tooth eruption
K00.7	Teething syndrome
K00.8	Other disorders of tooth development
K00.9	Disorder of tooth development, unspecified
**UMLS:ICD 10CM:K01**	**Embedded and impacted teeth**
K01.01	Embedded teeth
K01.02	Impacted teeth
**UMLS:ICD 10CM:K02**	**Dental caries**
K02.3	Arrested dental caries
K02.5	Dental caries on pit and fissure surface
K02.6	Dental caries on smooth surface
K02.7	Dental root caries
K02.9	Dental caries, unspecified
**UMLS:ICD 10CM:K03**	**Other disases of hard tissues of teeth**
K03.0	Excessive attrition of teeth
K03.1	Abrasion of teeth
K03.2	Erosion of teeth
K03.3	Pathological resorption of teeth
K03.4	Hypercementosis
K03.5	Ankylosis of teeth
K03.6	Deposits (accretions) on teeth
K03.7	Posteruptive color changes of dental hard tissues
K03.8	Other specified diseases of hard tissues of teeth
K03.9	Disease of hard tissues of tissues, unspecified
**UMLS:ICD 10CM:K04**	**Diseases of pulp and periapical tissues**
K04.0	Pulpitis
K04.1	Necrosis of pulp
K04.2	Pulp degeneration
K04.3	Abnormal hard tissue formation in pulp
K04.4	Acute apical periodontitis of pulpal origin
K04.5	Chronic apical periodontitis
K04.6	Periapical abscess with sinus
K04.7	Periapical abscess without sinus

K04.8	Radicular cyst
K04.9	Other and unspecified diseases of pulp and periapical tissues
**UMLS:ICD 10CM:K05**	**Gingivitis and periodontal diseases**
K05.0	Acute gingivitis
K05.1	Chronic gingivitis
K05.2	Aggressive periodontitis
K05.3	Chronic periodontitis
K05.4	Periodontitis
K05.5	Other periodontal diseases
K05.6	Periodontal diseases, unspecified
**UMLS:ICD 10CM:K06**	**Other disorders of the gingiva and edentulous alveolar ridge**
K06.0	Gingival recession
K06.1	Gingival enlargement
K06.2	Gingival and edentulous alveolar ridge lesions associated with trauma
K06.3	Horizontal alveolar bone loss
K06.8	Other specified disorders of gingiva and edentulous alveolar ridge
K06.9	Disorder of gingiva and edentulous alveolar ridge, unspecified
**UMLS:ICD 10CM:K08**	**Other disorders of teeth and supporting structures**
K08.0	Exfoliation of teeth due to systemic causes
K08.1	Complete loss of teeth
K08.2	Atrophy of edentulous alveolar ridge
K08.3	Retained dental root
K08.4	Partial loss of teeth
K08.5	Unsatisfactory restoration of teeth
K08.8	Other specified disorders of teeth and supporting structures
**UMLS:ICD 10CM:K09**	**Cysts of oral region, not elsewhere classified**
K09.0	Developmental odontogenic cysts
K09.1	Developmental (nonodontogenic) cysts of oral region
K09.8	Other cysts of oral region, not elsewhere classified
K09.9	Cyst of oral region, unspecified
**UMLS:ICD 10CM:K11**	**Diseases of salivary glands**
K11.0	Atrophy of salivary gland
K11.1	Hypertrophy of saliary gland
K11.2	Sialoadentitis
K11.3	Abcess of salivary gland
K11.4	Fistula of salivary gland
K11.5	Sialolithiasis
K11.6	Mucocele of salivary gland
K11.7	Disturbances of salivary secretion
K11.8	Other diseases of salivary glands
K11.9	Disease of salivary gland, unspecified
**UMLS:ICD 10CM:K12**	**Stomatitis and relation lesions**
K12.0	Recruitment of oral aphthae
K12.1	Other forms of stomatitis

K12.2	Cellulitis and abcess of mouth
K12.3	Oral mucositis (ulcerative)
**UMLS:ICD 10CM:K13**	**Other diseases of lip and oral mucosa**
K13.0	Diseases of lips
K13.1	Cheek and lip biting
K13.2	Leukoplakia of oral mucosa, including tongue
K13.3	Hairy leukoplakia
K13.4	Granuloma and granuloma-like lesions of oral mucosa
K13.5	Oral submucous fibrosis
K13.6	Irritative hyerplasia of oral mucosa
K13.7	Other and unspecified lesions of oral mucosa
**UMLS:ICD 10CM:K14**	**Diseases of tongue**
K14.0	Glossitis
K14.1	Geographic tongue
K14.2	Median rhomboid glossitis
K14.3	Hypertrophy of tongue papillae
K14.4	Atrophy of tongue papillae
K14.5	Plicated tongue
K14.5	Glossodynia
K14.8	Other diseases of tongue
K14.9	Other diseases of tongue, unspecified

These ICD identifiers have been established by the World Health Organization for the classification of medical conditions impacting the oral cavity and salivary glands.
